# Performance of Extracellular Vesicles From *Leishmania* (*Leishmania*) *infantum* for Serological Diagnosis of Human and Canine Visceral Leishmaniasis

**DOI:** 10.1155/japr/8355886

**Published:** 2025-01-21

**Authors:** Allecineia Bispo da Cruz, Francieli Marinho Carneiro, Noemi Nosomi Taniwaki, Gislene Mitsue Namiyama, Débora Oliveira dos Santos, Katia Gomes Castellão, Isabelle Martins Ribeiro Ferreira, Roberto Mitsuyoshi Hiramoto, Vera Lucia Pereira-Chioccola

**Affiliations:** ^1^Parasitology and Mycology Center, Adolfo Lutz Institute, Sao Paulo, Brazil; ^2^Graduate Program in Science, Coordinator for Disease Control, Ministry of Health of São Paulo State, Sao Paulo, Brazil; ^3^Electron Microscopy Center, Adolfo Lutz Institute, Sao Paulo, Brazil

**Keywords:** extracellular vesicles, human and canine visceral leishmaniasis, *Leishmania (L.) infantum*, serological diagnosis

## Abstract

Visceral leishmaniasis (VL) is a zoonotic disease in which dogs are the main reservoirs. Until now, the serological tests do not present satisfactory sensitivity for diagnosis of these hosts. One of the functions of extracellular vesicles (EVs) is related to immunological host response. Here, we evaluated the ability of EVs released by *Leishmania (Leishmania) infantum* promastigotes (Leish-EVs) to be source of antigens for use in serological diagnosis for human visceral leishmaniasis (HumVL) and canine visceral leishmaniasis (CanVL). A total of 300 sera were tested. The 155 human sera were divided into 4 groups and 145 canine sera into 3 groups. In human sera, Leish-EVs were reactive in 73/74 sera from patients with VL (Hum-VL) with 98.64% sensitivity. The 26 sera from healthy individuals (NH) and 27 from individuals with asymptomatic toxoplasmosis (ATx) were nonreagent (100% specificity). Leish-EVs-ELISA had cross-reactivity or inconclusive results in 13.5% of sera from Chagas disease patients (CD). In canine sera, Leish-EVs were reactive in 60/63 sera from dogs with CanVL (Can-VL) with 95.24% sensitivity. Leish-EVs were nonreactive in sera from 57 dogs without Can-VL (NC) and 25 with other infections (OIs) with 100% specificity. Hum-VL produced more IgG1 against Leish-EVs than IgG2, IgG3, and IgG4. Can-VL produced more IgG2 against Leish-EVs than IgG1. In conclusion, this study provides evidence that Leish-EVs released by *L. (L.) infantum* when used as antigen in ELISA identified the host antibodies. The methodology was effective for serological diagnosis of VL, since results exhibited good sensitivity and specificity for human and canine sera.

## 1. Introduction

Visceral leishmaniasis (VL) is a life-threatening disease caused by protozoans from *Leishmania (Leishmania) donovani* complex. This infection is one of the most severe forms of leishmaniasis, since it compromises internal organs and causes high lethality in untreated hosts. Since its severity, VL is one of the top 10 neglected tropical diseases affecting a large proportion of people and dogs that are, indirectly, the principal transmission source [1–5].

VL is endemic in around 80 countries with high mortality rates. Worldwide, VL affects more than 12 million people. Around 1.5 million of new cases occur per year, and approximately 1 billion people are at risk to infect them [4–6]. VL is endemic in the Americas, Africa, Southern Europe, and Asia and in Brazil accounts for 90% of cases in Latin America [1–8]. The infections are caused by *Leishmania (Leishmania) infantum* and the transmission occurs by the bite of the sand flies, of which the main vector species is *Lutzomyia longipalpis* [7–12].

The sand fly bite transmits the promastigotes, which transform into amastigotes in the host and multiply by binary division in the mononuclear phagocytic system [12, 13]. Infected individuals have long periods of fever, weight loss, anemia, and changes in internal organs such as the spleen and liver, leading to progressive weakness and progression to death. This cycle can vary according to the geographical region, *Leishmania* species, and invertebrate and vertebrate hosts [13, 14].

Among wild and urban animals, the dogs are the main reservoirs of VL due to their close association with humans and their great epidemiological importance in rural and urban areas [9, 15]. Canine visceral leishmaniasis (CanVL) is a severe disease and potentially fatal. The most frequent clinical signs are lymphadenopathy, onychogryphosis, cutaneous lesions, weight loss, cachexia, fever anemia, and locomotor abnormalities [9, 16, 17].

Several serological tests have been evaluated for use in laboratory diagnosis, epidemiological studies, control programs, and field studies. The most used tests are indirect immunofluorescence assay (IFAT), enzyme-linked immunosorbent assay (ELISA), direct agglutination (DAT), and immunochromatography [18–20]. However, none of them have high sensitivity (Sen) and specificity (Spe). At the same time, a variety of antigens have been tested for use in ELISA including crude soluble antigens, recombinant *Leishmania* proteins, and kinesin-derived proteins such as rK39, rKDDR, and rKDDR-plus [19–21]. These targets have good discriminatory ability but, sometimes, the diagnostic performance is varied [20, 21]. Since VL is a killer disease with a significant impact on livelihoods such as agriculture and livestock and, more recently, in big cities, the accurate diagnosis is very important to give a rapid and appropriate treatment.

Studies on extracellular vesicles (EVs) have been growing interest in protozoan parasitic diseases. EVs are a group of membrane-enclosed nanoparticles that are released to extracellular space by all cells [22–24]. There are different types of EVs, and they are classified based on size and composition. The largest are the apoptotic bodies that arise from the outward fragments of apoptotic cells, resulting in a 500–5000 nm in diameter phosphatidylserine-rich vesicles. The microvesicles are particles of 100–1000 nm in diameter that are formed from the plasma membrane and are enriched with phosphatidylserine and cholesterol. The exosomes are the littlest EVs (30–150 nm) formed by the exocytosis of multivesicular bodies. They are released after the fusion with the plasma membrane [23–26]. Cells use EVs as mediators of the intracellular communication, as well as the transport of proteins, lipids, and nucleic acids to short- and long-distance cells [27, 28]. The EV release by *Leishmania* is a natural mechanism employed during the invasion of the host cells. Thus, EVs play a crucial role in the first moments of infection, promoting the parasite survival, increasing the parasite virulence, and causing a worsening of the overall disease picture by immune suppressing the host [29].

Proteomic studies have found different proteins in leishmanial exosomes. However, GP63 is one of the principal *Leishmania* virulence factor, since it is a zinc-dependent metalloprotease present in great quantities on the surface of the promastigote forms. Around 1% of the entire parasite proteome is anchored via glycophosphatidylinositol (GPI) anchor [30–33]. In addition, EVs produced by *Leishmania* are highly antigenic for a human promonocyte cell line, causing an increase of IL-10 and IL-12 [29].

Although the majority of these studies were performed analyzing EVs produced by parasites cultivated in axenic cultures, our group previously described that sera from dogs with CanVL (Can-VL) are reactive against *L. (L.) infantum* EVs (Leish-EVs) [34]. Joined with these studies, due to the ease of producing EVs and the ability of these nanoparticles to be highly immunogenic, this study evaluated whether Leish-EVs could be a good source of antigens for use in serological diagnosis of VL.

## 2. Material and Methods

### 2.1. Serum Samples

The capability of Leish-EVs to react with serum antibodies from Hum-VL (patients with VL) and Can-VL was evaluated employing 300 sera.

For the human diagnosis, 155 sera were tested divided into four groups as follows: (1) Hum-VL (74 sera), (2) NH (normal individuals) (26 sera), (3) ATx (individuals with asymptomatic toxoplasmosis) (27 sera), and (4) CD (patients with Chagas disease) (28 sera). Patients or individuals from Groups 2, 3, and 4 lived in nonendemic areas for leishmaniasis. Results of sera from Group 2 (NH) were used to calculate the cutoff and Spe.

For the canine diagnosis, 145 canine sera were tested and divided into three groups as follows: (1) Can-VL (63 sera); (2) NC (normal dogs—without CanVL) (57 sera), this group was composed of 21 sera from dogs that lived in endemic areas for leishmaniasis and 36 sera from dogs that lived in nonendemic areas for leishmaniasis; and (3) OI (other infection) (25 sera from dogs with other canine diseases, such as toxoplasmosis (5 sera), ehrlichiosis (10 sera), leptospirosis (5 sera), and spotted fever (5 sera)). The results of the 57 sera from Group 2 (NC) were used to calculate the cutoff and Spe. The sera from human Groups 3 and 4 and from canine Group 3 were included in the study since they had infections that can have cross-reaction with VL.

All clinical samples used in this study were sent to Instituto Adolfo Lutz, within 48 h after collection and immediately processed for molecular and/or serological tests. Next, they were stored at −20°C until use in this study. The clinical samples of patients or individuals were forwarded from different hospitals of São Paulo State. Those from dogs were collected by veterinarians in municipalities in small municipalities close to each other in the northwest region of São Paulo State, Brazil.

### 2.2. Selection of Human and Canine Sera

The serological diagnosis for HumVL included (a) “*Leishmaniose VH Bio, Human, K218*” (Bioclin) and (b) a rapid immunochromatographic test for qualitative detection of human IgG anti-*Leishmania* spp. (*L. (L.) donovani*, *Leishmania (Leishmania) chagasi*, and *L (L.) infantum)* [35, 36]. For Chagas disease, the serological diagnosis was performed by (a) “*BioLISA Chagas recombinante K180*” (Bioclin) and (b) “*IFI Chagas Bio-Manguinhos*” produced by Bio-Manguinhos (FIOCRUZ). For toxoplasmosis, the serological diagnosis was performed by *Anti-Toxoplasmose ELISA, IgG and IgM* (Euroimmun).

The serological diagnosis for CanVL included (a) “Dual Path Platform (DPP) CanVL” that is based on a recombinant protein rK28, a chimera that combines the K9, K26, and K39 antigens, from *L. (L.) infantum*, and (b) ELISA, produced by Bio-Manguinhos (FIOCRUZ). The serological diagnosis for canine toxoplasmosis was performed by Biolisa Toxoplasmose IgG and IgM (Bioclin), and the serological diagnosis for ehrlichiosis was performed by the kit *Erliquiose IgG vet fast* (Bioclin Vet) and for leptospirosis by the Kit *Canine Leptospira (ImmunoComb*), and for spotted fever, the tests were performed by “*in-house”* indirect immunofluorescence reaction.

### 2.3. Molecular Detection of *L. (L.) infantum* and *Trypanosoma cruzi*

The molecular detection of *L. (L.) infantum* and *T. cruzi* was performed by real-time PCR (qPCR) as described before [37, 38], respectively. Briefly, plasma samples were separated by centrifugation. Pellets were mixed with three times the volume of a buffer containing 150-mM ammonium chlorate, 1-mM potassium bicarbonate, and 0.1-mM EDTA, pH 7.3, incubated for 15 min at room temperature under mild shaking and centrifuged (2500*g*/10 min). DNA molecules were extracted by QIAamp DNA Mini Kit (Qiagen). The protocols were followed according to the manufacturer's instructions in a robotic workstation for automated purification of DNA (QIAcube, Qiagen). DNA concentrations and purity were determined by the ratio of optical density (OD) at 260 and 280 nm in NanoDrop One and diluted with ultrapure water until concentration 100 ng/*μ*L.

*L. (L.) infantum* (belonging to *L. donovani* complex) was identified by qPCR. The design included the forward Ldon37act-F (5⁣′AAGTGCGACATTGATGTGCGC3⁣′) and reverse Ldon114act-R (5⁣′AAGGTTGAGGAACATGGTCGAC3⁣′) primers and the hybridization probe Ldon66act-PB (5⁣′CCGGACAGCACGATGTTCCCGTAC3⁣′) labeled with FAM (6-carboxyfluorescein) and BHQ1 (Black Hole Quencher 1) at the 5⁣′ and 3⁣′ ends, respectively. *T. cruzi* was identified using the Cruzi 32F and Cruzi 148R primer set and probe 71 labeled with FAM and BHQ1 at 5⁣′ and 3⁣′ ends, respectively.

The reactions were performed in the final volume of 20 *μ*L. DNA samples and controls (3 *μ*L) were added to reaction mixture containing 10 *μ*L of 2X TaqMan Universal PCR Master Mix and 1.25 *μ*L of “Assay Mix” (18 *μ*M of forward and reverse primers and 5 *μ*M of the hydrolysis probe). Amplifications were performed in an AriaMx-Real Time PCR System (Agilent Technologies) using the following thermal profile: one cycle at 95°C for 2 min, next, 40 cycles at 95°C for 3 s, and one cycle for 60°C for 20 s. Each amplification reaction included a negative control (ultrapure water) and a positive control (DNA extracted from each parasite species). The absence of reaction inhibitors was determined by amplification of the constitutive gene in mammals (RNAse).

### 2.4. *L. (L.) infantum* Cultures, Production of Leish-EVs, and Transmission Electron Microscopy (TEM)

The protocols were performed as described before [29]. Promastigotes of *L. (L.) infantum* (MHOM/BR/1972/LD) were maintained at 25°C, in 199 medium, pH 7.2 containing fetal bovine serum 10%, gentamicin 30 *μ*g/mL, hemin 50 *μ*L/mL, and human urine 50 *μ*L/mL.

For Leish-EV purification, promastigotes were collected from culture medium in the log phase of growth, counted, concentrated by centrifugation (2500 × *g* for 15 min), and washed (5 times) with sterile phosphate-buffered saline (PBS). Next, parasites were resuspended in RPMI medium (1 mL) and incubated for 2 h at 37°C to release EVs in the culture medium. Afterward, the mixture was centrifuged (in the same conditions) and filtered (0.22 *μ*m) to remove the parasites. The supernatant containing Leish-EVs was treated with 10 *μ*g/mL of a cocktail of protease inhibitors containing per milliliter 20 *μ*M AEBSF (4-(2-aminoethyl)benzenesulfonyl fluoride hydrochloride), 10 *μ*M EDTA, 1.3 *μ*M bestatin, 0.14 *μ*M E-64, 10 nM leupeptin, and 3 nM aprotinin (Sigma-Aldrich). Thereafter, each aliquot was stored at −70°C until analysis.

For use as antigen in immunoblot, dot blot, and ELISA, Leish-EVs dissolved in RPMI (1 mL) were transferred to Ultra-Clear centrifuge tubes (6 mL tube for SW-55 rotor) (Beckman Coulter, Brea, California, United States) and complete the volume (6 mL) with filtered PBS and ultracentrifuged at 100,000 × *g* for 60 min at 25°C in a Beckman Coulter L8-80M centrifuge. The pellet containing Leish-EVs was suspended in 100 *μ*L of filtered PBS containing the cocktail of protease inhibitors. Next, the protein concentrations of Leish-EVs were determined by BCA (bicinchoninic acid) protein kit (Pierce) according to the manufacturer's instructions and by a NanoDrop One spectrophotometer (Thermo Fisher Scientific) at 280 nm. For TEM, Leish-EVs were fixed in 2% paraformaldehyde/PBS (*v* : *v*) for at least 60 min. One drop of the suspension was put on an EM grid and performed by negative staining technique with 2% potassium phosphotungstate at pH 6.8. Grids were observed under a JEOL Transmission Electron Microscope (model JEM1011; JEOL/Massachusetts, USA) operating at 80 kV. Images were recorded with a Gatan 785 ES1000W Erlangshen camera.

### 2.5. Immunoblot and Dot Blot

The aim of the experiments including immunoblot and dot blot was to show the reactivity of Leish-EVs with the sera from infected hosts [29]. Leish-EVs were solubilized in lysis buffer (SDS 2%, glycerol 10%, 2-mercaptoethanol 5%, 60 mM Tris–HCl, pH 6.8, and bromophenol blue 0.002%), boiled, and run in 12% polyacrylamide-SDS gels. The proteins of Leish-EVs were seen after silver staining. For immunoblot, Leish-EV proteins were transferred at a constant voltage of 15 V for 30 min to nitrocellulose membranes (Bio-Rad).

For dot blots, strips of nitrocellulose membranes (8 mm × 40 mm) with 0.45 *μ*m in diameter were incubated with Leish-EVs (1.5 *μ*g/mL) and washed five times with PBS during 5 min. Next, the free binding sites in dot blot membranes and immunoblot were blocked with 5% skim milk–PBS for 60 min at room temperature. After washing (three times with PBS), membranes were incubated for 18 h (4°C) with the human and canine serum samples, with and without VL, diluted 1:50 in 5% skim milk–PBS. After washing (three times), the membranes were incubated at room temperature (120 min) with the secondary antibody, a peroxidase-conjugated rabbit anti-dog IgG diluted 1:4000 in 5% skim milk–PBS (Sigma Aldrich) or a peroxidase-conjugated goat anti-human IgG (Sigma Aldrich) diluted 1:500 in 5% skim milk–PBS. Bound antibodies from dot blots and immunoblotting were visualized after treatment with chemiluminescence western blotting substrate (Pierce ECL Western Solution, Thermo Scientific). The images were captured on documenting gel with a chemiluminescence filter (UVITEC, Cleaver Scientific) in a Blot Scanner (C-DiGit).

### 2.6. Leish-EVs-ELISA: Standardization and Diagnostic Performance

For Leish-EVs-ELISA, 96-well plates (flat bottom, medium binding, Costar or Jet-Biofil) were sensitized 18 h at 4°C with Leish-EVs dissolved in 0.1 mL of 0.1 M NaHCO_3_, pH 8.5. For standardizations, the concentrations of 0.5, 1.0, 2.0, and 3.0 *μ*g/mL were tested. Unbound antigens were removed by washing the plates (two times) with 0.05% Tween 20–PBS. The free binding sites were blocked with 5% skim milk–PBS for 60 min at 37°C. Next, plates were washed for another five times with 0.05% Tween 20–PBS. For standardization, serum samples diluted at 1:100, 1:150, and 1:200 in 5% skim milk–PBS were tested. The serum samples were applied, in duplicate and incubated for 60 min at 37°C. Then, the plates were rewashed with 0.05% Tween 20–PBS (three times) and incubated for another 60 min at 37°C with a peroxidase-conjugated rabbit IgG anti-dog IgG (Sigma Aldrich) or a peroxidase-conjugated goat IgG anti-human IgG (Sigma Aldrich) diluted 1:20,000; 1:22,000; and 1:25,000 in 5% skim milk–PBS. After another wash cycle for reaction development, 100 *μ*L/well of TMB/H_2_O_2_ chromogen was added in the plates and different times of incubation were tested (10, 15, and 20 min) at room temperature protected from light. The reactions were stopped by adding 50 *μ*L of H_2_SO_4_ 4 N and the absorbance was measured with an ELISA reader LMR-96 (Loccus) with a 450–630 nm filter. All samples were tested twice to ensure accuracy (AC), and the results were recorded in duplicate. Two controls were included on each plate: the positive, which consisted of 50 *μ*L of known reagent serum, and the negative, with a nonreactive serum sample (diluted under the same conditions).

The NH group (26 sera) was tested in Leish-EVs-ELISA for human samples and the NC group for canine samples. The cutoff of each Leish-EVs-ELISA (for dog or human) corresponded to the average OD (450–630 nm) of the negative group ± 2 standard deviation (SD). The grey zone corresponded to 10% upper to OD values of cutoff. Samples with OD in this range were considered inconclusive.

The OD results were transformed to Leish-EVs-ELISA-relative values and are shown in the figures. The Leish-EVs-ELISA-relative values represent the ratio of the absorbance of each serum sample at an optical density of 450–630 nm to the cutoff value—(serum OD/cutoff OD) Values greater than 1.0 were considered reactive [39–41].

### 2.7. Human and Canine IgG Subclasses

The aim of the study of levels of IgG subclasses (canine and human) was to know which of them were reactive against Leish-EVs. The IgG subclasses for dogs and humans were determined by ELISA using 15 sera for each group (Hum-VL, NI, Can-VL, and ND). The human IgG subclasses were determined using four secondary polyclonal antibodies (Sigma), horseradish peroxidase–conjugated goat IgG anti-human-IgG1 at dilution 1:500 and anti-IgG2, anti-IgG3, and anti-IgG4 that were used at dilution 1:1000 as previously described [42]. The two secondary polyclonal antibodies for dogs were goat IgG anti-dog-IgG1 and sheep IgG anti-dog-IgG2. Both were horseradish peroxidase–conjugated (Bethyl). The standardization of the secondary antibodies consisted in dilutions of 1:5000; 1:10,000; 1:15,000; and 1:20,000. The results of each IgG subclass were shown as OD at 450–630 nm.

### 2.8. Statistical Analyses

The statistical analyses were evaluated using GraphPad Prism Software Version 8.0 for Windows (San Diego, California, United States). Comparisons between groups including the analysis of the two canine IgG classes were calculated by a two-tailed unpaired test. The analysis of the four human IgG classes was calculated by the ordinary one-way ANOVA. The differences or similarities were considered statistically significant when *p* ≤ 0.05. The receiver operating characteristic (ROC) curves were constructed to compare the groups Hum-VL versus NH (for human sera) and Can-VL versus NC (for canine sera). The results generated positive predictive value (PPV), negative predictive value (NPV), AC, Sen, Spe, area under the ROC curve (AUC), and 95% confidence intervals.

## 3. Results

### 3.1. Leish-EVs-ELISA: Standardization and Serological Diagnosis of the Human and Canine Sera

The results of the standardization of Leish-EVs-ELISA included the following: (a) the antigen concentration (Leish-EVs) for the plate sensitization was 2.0 *μ*g/mL; (b) the serum dilution was 1:200; (c) the concentration of the secondary antibodies for canine samples: for rabbit IgG anti-dog IgG it was 1: 22,000; for goat IgG anti-dog IgG1 it was 1:5000; for sheep IgG anti-dog-IgG2 it was 1:15,000. For human samples, the goat IgG anti-human IgG was 1:20,000; (d) the chromogen time was defined as 20 min.

Next, Leish-EVs were tested as antigen in ELISA to assess the capacity to be used in the serological diagnostic for HumVL and CanVL. The chosen human and canine sera were based on results from serological, molecular, and clinical diagnosis described in Tables 1 and 2.

Table 1 shows the results of human sera for serological diagnosis performed with commercial kits and qPCR for *L. (L.) infantum* and *T. cruzi*.  Table 2 shows the results of canine sera for serological diagnosis performed with commercial kits and for qPCR for detection of *L. (L.) infantum*.

### 3.2. Study of Leish-EVs From *L. (L.) infantum*

**Figure 1:**
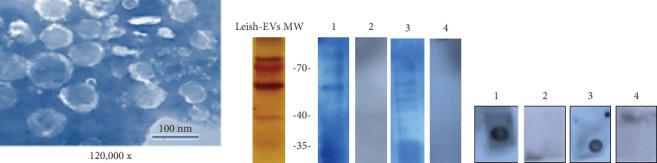
Biological characteristics and reactivity of Leish-EVs. (a) Image capitated by TEM showing Leish-EVs released by promastigotes containing microvesicles and exosomes. (b) Electrophoretic analysis of Leish-EVs on 12% SDS-PAGE stained with silver nitrate (Strip 1). Leish-EVs were used as antigen in immunoblot and tested in two canine sera: one reagent (Strip 2) and another nonreagent (Strip 3) for CanVL. Strips 3 and 4 show the reaction of two human sera: one reagent and another nonreagent, respectively, for VL. (c) Leish-EVs were used as antigen in dot blot tested the same sera than those of (b).

Leish-EVs released by promastigotes in culture medium at 25°C for 2 h are shown in Figure 1(a). The image acquired by TEM shows many exosomes and microvesicles. At the same time, Figure 1(b) shows the electrophoretic profile of Leish-EVs on 12% SDS-PAGE after the silver nitrate stain. Next, these nanoparticles were used as antigen in the immunoblots testing 4 sera (2 from dogs and 2 from humans). Strip 1 was reactive and Strip 2 nonreactive for CanVL. In the same way, Strip 3 was reactive and Strip 4 nonreactive for HumVL. These results also were confirmed in dot blot (Figure 1(c)).

### 3.3. Performance of Leish-EVs Tested as Antigen in ELISA

**Figure 2:**
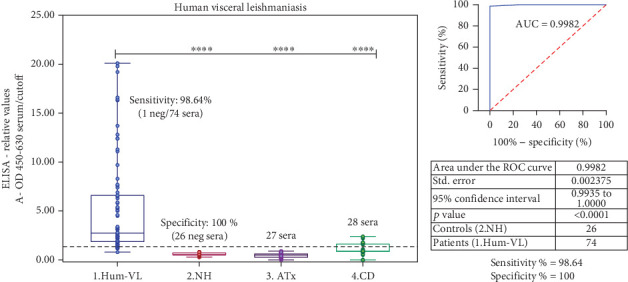
Performance of Leish-EVs-ELISA for use in serological diagnosis of human VL. (a) ELISA-relative values of 155 human sera obtained by Leish-EVs-ELISA. Four groups of sera were formed: (1) Hum-VL (74 patients with VL), (2) NH (26 healthy individuals), (3) ATx (27 individuals with asymptomatic toxoplasmosis), and (4) CD (28 patients with Chagas disease). Values are expressed as mean ± SEM of ELISA-relative values. The Hum-VL group was statistically different at ⁣^∗∗∗∗^*p* < 0.0001 from NH, ATx, and CD groups. (b) ROC curve was constructed from ELISA-relative values of Leish-EVs-ELISA from the Hum-VL group (74 sera/patients with VL) and the NH group (26 sera/individuals without VL).

Figure 2 shows results of Leish-EVs-ELISA for Hum-VL.  Figure 2(a) shows the distribution of the 155 human sera. Leish-EVs were reactive in 73 of the 74 sera from patients with VL (Hum-VL group), with 98.64% Sen. The 26 sera from healthy individuals living in nonendemic leishmaniasis areas (NH group) had nonreagent results with 100% Spe. However, Leish-EVs-ELISA had 13.5% cross-reactivity or inconclusive results in the CD group. The statistical analyses by the two-tailed unpaired test revealed that the Hum-VL group was statistically different from the NH, ATx, and CD groups at *p* < 0.0001.  Figure 2(b) shows an excellent ROC curve with the cutoff and Sen (Hum-VL group) different from the NH group and AUC = 0.9982. The analyses also determined 100% PPV, 96.29% NPV, and 99.00% AC (Table 3).

**Figure 3:**
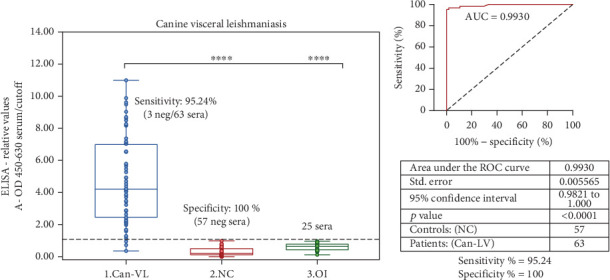
Performance of Leish-EVs-ELISA for use in serological diagnosis of CanVL. (a) ELISA-relative values of 145 canine sera obtained by Leish-EVs-ELISA. Three groups of sera were formed: (1) Can-VL (63 dogs with CanVL), (2) NC (57 dogs without CanVL), and (3) OI (25 dogs with other canine diseases). Values are expressed as mean ± SEM of ELISA-relative values The Can-VL group was statistically different at ⁣^∗∗∗∗^*p* < 0.0001 from the other two groups. (b) ROC curve of Leish-EVs-ELISA constructed from ELISA-relative values of the Can-VL group (63 sera from dogs with CanVL) and the NC group (57 sera without CanVL).

In parallel, Figure 3 shows the results of Leish-EVs-ELISA for CanVL.  Figure 3(a) shows the distribution of 145 canine sera. Leish-EVs were reactive in 60 of the 63 sera from the Can-VL group with 95.24% Sen. The NC group had nonreagent results in 57 sera with 100% Spe. In the same way, the OI group had nonreagent results in the 25 sera from dogs with other infections (toxoplasmosis, ehrlichiosis, leptospirosis, and spotted fever). The Can-VL group was statistically different from the other groups at *p* < 0.0001, by two-tailed unpaired test, just like Leish-EVs-ELISA tested in human sera.  Figure 3(b) shows the ROC curve produced by Leish-EVs-ELISA tested in canine sera with *AUC* = 0.9930. Both ROC curves proved the particularly good results of Leish-EVs-ELISA.  Table 3 shows the values of 100% PPV, 95.00% NPV, and 97.50% AC.

### 3.4. Levels of IgG Subclass Produced Against Leish-EVs

**Figure 4:**
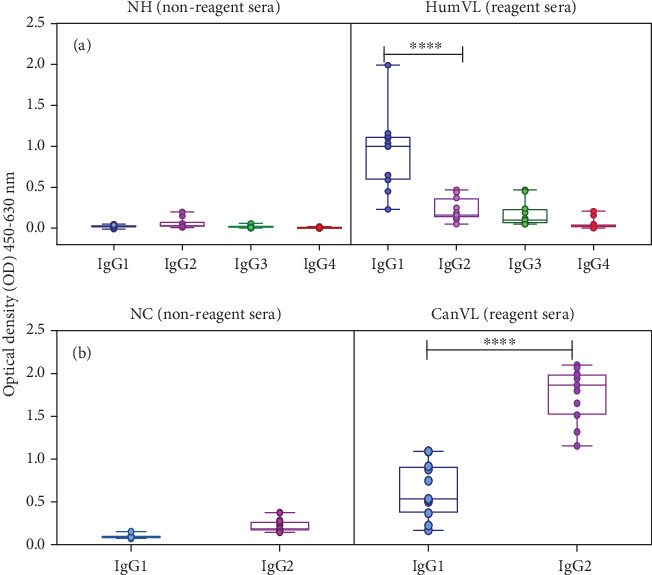
Immunological reactivity in human and canine sera for IgG subclass against Leish-EVs. (a, b) IgG subclass against Leish-EVs determined by Leish-EVs-ELISA in human sera. The results were expressed as OD mean ± SE from each group. IgG1 levels were statistically different from IgG2, IgG3, and IgG4 at ⁣^∗∗∗∗^*p* < 0.0001.

In the next step, the levels of human IgG subclass produced against Leish-EVs were evaluated by Leish-EVs-ELISA and the values were expressed as OD mean ± SE. For IgG1 of the NH group, the mean values were 0.023 ± 0.004; for IgG2, 0.050 ± 0.014; for IgG3, 0.023 ± 0.004; and for IgG4, 0.006 ± 0.002. In contrast, in the Hum-VL group, the OD mean values were 0.967 ± 0.126 for IgG1, 0.229 ± 0.038 for IgG2, 0.179 ± 0.040 for IgG3, and 0.067 ± 0.019 for IgG4. The IgG1 levels of the Hum-VL group was statistically different from those of the other groups at *p* < 0.0001, by one-way ANOVA (Figure 4(a)). At the same time, the canine IgG subclasses produced against Leish-EVs were evaluated by Leish-EVs-ELISA. For IgG1 of the NC group, the OD mean values were 0.095 ± 0.006 and for IgG2 0.218 ± 0.021. In contrast, in the Can-VL group, the OD mean values were 0.640 ± 0.098 for IgG1 and 1.789 ± 0.100 for IgG2. The IgG1 levels from the Can-VL group were statistically different from the IgG2 levels, at *p* < 0.0001 by two-tailed unpaired Student's *t*-test (Figure 4(b)).

## 4. Discussion

VL represents an important threat to the millions of individuals living in endemic regions. Global warming can widen the geographical spread of sand fly leading to more people living at risk of contracting this disease. As there is lack of vaccines for preventing this infection and symptoms similar to other diseases, it is extremely important for an accurate diagnosis for patients to receive the appropriate treatment [43]. The relationship between EVs and pathogenic protozoa is an important factor for parasite survival. As evidenced in parasitological diseases, EVs influence the immunity of infected mammals [29, 34, 43–45]. This situation is probably promoted by the higher release of Leish-EVs by *L. (L.) infantum* promastigotes in mammals than in vectors [29, 46, 47]. Other studies, also, suggested that a good quantity of Leish-EVs released by parasites facilitates their penetration in mammal macrophages [48, 49]. The Leish-EVs, still, assist *Leishmania* species in adapting to the host environment, carrying virulence factors that affect the modulation of the cellular immune responses [49, 50]. Corroborating with these data, our previous studies and this study show by immunoblot and dot blot that sera from patients and dogs with VL are reactive against Leish-EVs [29, 34].

Various factors can increase the spread of leishmaniasis in different areas of the world. Dogs, the main reservoirs for VL, have an intense relationship with men, since they are their pets. This intense relationship, principally in the last years, caused increased travel and dog importation [9]. By the biological characteristics of *Leishmania* spp., the diagnostic tests still show low Spe and cross-reactivity with other pathogens, such as *Ehrlichia canis*, *Babesia canis*, *Toxoplasma gondii*, and *T. cruzi* [51–54]. These factors have been leading the investigation of other forms of antigens to use in serological diagnosis. Based on these data and the importance of the diagnostic tests for control programs and surveillance of infected dogs, this study was aimed at evaluating whether Leish-EVs could be used as antigen.

The standardization of the methodologies improved so much the result evaluation. In addition, Leish-EVs-ELISA was always tested using a well-characterized panel of human samples, enabling good results as 98.64% Sen, 100% Spe, and 99.00% AC. Similar results were shown when the panel of canine sera was tested. Leish-EVs-ELISA using dog sera presented 95.24% Sen, 100% Spe, and 97.50% AC. Both ROC curves confirmed the excellent results.

The performance of the serological diagnosis for VL may vary depending on the host factors and the geographic region where the infection was acquired [51–56]. Variations in Sen and Spe might occur when antigens are tested in samples from different localities [53–56]. The validation of Leish-EVs-ELISA for HumVL was performed using sera from patients with VL, toxoplasmosis, and Chagas disease and healthy individuals. For CanVL, the sera were from dogs with VL, ehrlichiosis, toxoplasmosis, leptospirosis, and spotted fever and healthy dogs that lived in endemic and nonendemic areas.

A global study obtained lower Sen in East Africa and Brazil, when compared with an Indian subcontinent region. However, in other studies, the rK39-ELISA test exhibited 82% Sen [56]. The AC obtained in human sera in this study was superior to those of the routine tests used in HumVL diagnosis in Brazil [57]. *Leishmani*a–ELISA platform that includes ELISA IgG + IgM, Ridascreen Ab, and NovaLisa–*Leishmania infantum*–IgG presented an AC of 85.5%–91.2%.

The occurrence of visceral and tegumentary leishmaniasis in different Brazilian regions, as well as Chagas disease, coupled with the phylogenetic proximity of their infectious agents causes the occurrence of cross-reactions in sera from patients with these diseases. In this study, the results of Leish-EVs-ELISA reveal 13.5% cross-reactivity and results in grey zone were shown in sera from Chagas disease patients. Previous studies reported similar results. rK39-ELISA had 85% cross-reactivity for malaria and 83.3% for Chagas disease [58]. Another study reported that 25–50.8% cross-reactions occurred in sera from patients with acute and chronic Chagas disease and patients with cutaneous or mucosal forms of leishmaniasis in an ELISA using *L. chagasi* exoantigens [59]. In another study, the *L. infantum* exoantigen had 35% cross-reactivity in sera of patients with Chagas disease [60]. On the other hand, a Brazilian multicenter study demonstrated that a chimeric *T. cruzi* antigen used in ELISA produced cross-reactivity in sera from humans and dogs with visceral and mucocutaneous leishmaniasis. These sera were from hosts that lived in coendemic areas [61–63]. Thus, it is reasonable to conclude that the immunoreactivity of Leish-EVs-ELISA observed in this study occurred due to the antigenic similarities among the species of family Trypanosomatidae. Certainly, Leish-EVs carry similar antigens of both species.

Concerning Leish-EVs-ELISA for CanVL, the performance presented excellent results with 95.24% Sen, 100% Spe, and 97.50% AC. No cross-reactions were observed in the OI group (25 sera of dogs with toxoplasmosis, ehrlichiosis, leptospirosis, and spotted fever). These results indicated that Leish-EVs-ELISA is able to discriminate VL from the other widely distributed diseases in Brazil. In addition, no difference was observed in ELISA-relative values in canine sera from endemic or nonendemic areas (NC). Similar results were shown in other studies. rKR95-ELISA exhibited 95.9% Sen and 96.4% Spe in canine sera [64]. However, the Kalazar Detect test (rK39 antigen) exhibited cross-reactivity with *E. canis*, *T. gondii*, and *Neospora caninum* [65, 66].

The investigations of IgG subclasses demonstrated that VL patients produced much more IgG1 against Leish-EVs when compared with the other IgG subclasses (IgG2, IgG3, and IgG4). Studies have shown that IgG1 production is high in patients with the active disease. All sera tested in this study were from patients admitted in hospitals for VL diagnosis and posterior treatment. After treatment, patients began to produce IgG2 antibodies [67–70]. Regarding Can-VL, the results demonstrated that they produced more IgG2 against Leish-EVs than IgG1. These data are in accordance with our previous study that demonstrated that Leish-EVs stimulate the immune response, contributing to immunosuppression and immunopathology in infected hosts [29]. Nevertheless, the dog results were not compared with other studies since, among them, conflicting results were reported including different levels of specific IgG1 and IgG2 for different antigens or reactions [70–74].

In summary, this study provides evidence that host antibodies recognize the Leish-EVs released by *L. (L.) infantum* as shown in Leish-EVs-ELISA. The methodology was effective for serological diagnosis of VL, since results exhibited good Sen and Spe for human and canine sera. In addition, the recovery of these nanoparticles is relatively simple for use as antigen.

## Figures and Tables

**Table 1:** Identification of human sera used in this study.

**Group (number of samples)**	**Human groups**			
	**Hum-VL** ^**a**^ ** (74)** ^**#**^	**NH** ^**b**^ ** (26)**⁣^∗^	**ATx** ^**c**^ ** (27)**⁣^∗^	**CD** ^**d**^ ** (28)**⁣^∗^
Molecular diagnosis (qPCR)	Positive for *L. (L.) infantum* (67)	*nd * ^e^	*nd*	Positive for *T. cruzi* (15)
Clinical diagnosis	Patients admitted in hospital for VL treatment (74)	Healthy (26)	Asymptomatic toxoplasmosis (27)	Patients with Chagas disease and immunosuppression (15) Patients with chronic Chagas disease (13)
Serological diagnosis				
Visceral leishmaniasis	Reagent (74)	Nonreagent (26)	Nonreagent (27)	Nonreagent (28)
Toxoplasmosis	*nd*	*nd*	Reagent (27)	*nd*
Chagas disease	*nd*	*nd*	*nd*	Reagent (28)

^a^Sera from HumVL, patients with VL.

^b^Sera from NH, normal individuals (without HumVL).

^c^Sera from ATx, individuals with asymptomatic toxoplasmosis.

^d^Sera from CD, patients with Chagas disease.

^e^ *nd*, nondetermined.

^#^Patients that lived (or live) in an endemic region for VL.

⁣^∗^Individuals/patients that never lived in endemic areas for leishmaniasis.

**Table 2:** Identification of canine sera used in this study.

	**Canine groups**		
**Group (number of samples)**	**Can-VL** ^**a**^ ** (63)** ^**#**^	**NC** ^**b**^ ** (57)** ^**#**^⁣^∗^	**OI** ^**c**^ ** (25)**⁣^∗^
Molecular diagnosis (qPCR)	Positive for *L. (L.) infantum* (50)	*nd * ^d^	*nd*
Clinical diagnosis	CanVL—with characteristic symptoms (63)	Healthy (57)	Other canine diseases
Serological diagnosis for visceral leishmaniasis	Reagent (63)	Nonreagent (57)	Nonreagent (25)
Serological diagnosis for other diseases	*nd*	*nd*	Reagent⁣^∗∗^ (25)

^a^Sera from Can-VL, dogs with CanVL.

^b^Sera from NC, dogs without CanVL.

^c^Sera from OI, dogs with other canine diseases.

^d^ *nd*, nondetermined.

^#^Dogs that lived in an endemic region for VL, including 21 dogs from the NC group.

⁣^∗^Dogs that never lived in endemic areas for leishmaniasis.

⁣^∗∗^Five sera for toxoplasmosis, 10 sera for ehrlichiosis, 5 sera for leptospirosis, 5 sera for spotted fever.

**Table 3:** Performance of Leish-EVs-ELISA for serological diagnosis of visceral leishmaniasis in humans and dogs.

**Group of sera (reactive/total)**	**Sen (%)**	**Spe (%)**	**PPV (%)**	**NPV (%)**	**AC (%)**
Hum-VL (73/74)	98.64	100	100	96.29	99.00
Can-VL (60/63)	95.24	100	100	95.00	97.50

Abbreviations: AC, accuracy; NPV, negative predictive; PPV, positive predictive value; Sen, sensitivity; Spe, specificity.

## Data Availability

The datasets supporting the results and conclusion of this article were included within the manuscript file. The original dataset can be given upon request.

## References

[1] Matsumoto P. S. S., Hiramoto R. M., Pereira V. B. R. (2021). Impact of the dog population and household environment for the maintenance of natural foci of *Leishmania infantum* transmission to human and animal hosts in endemic areas for visceral leishmaniasis in Sao Paulo state, Brazil. *PLoS One*.

[2] WHO (2010). *Control of the Leishmaniases*.

[3] WHO (2021). Leishmaniasis, number of cases of visceral leishmaniasis reported. http://apps.who.int/neglected_diseases/ntddata/leishmaniasis/leishmaniasis.html.

[4] Wamai R. G., Kahn J., McGloin J., Ziaggi G. (2020). Reviewer recognition and thanks. *Journal of Global Health Science*.

[5] Fernández-Prada C., Douanne N., Minguez-Menendez A., Kunal R. (2019). Repurposed molecules: a new hope in tackling neglected infectious diseases. *In Silico Drug Design: Repurposing Techniques and Methodologies*.

[6] WHO Leishmaniasis. https://www.who.int/health-topics/leishmaniasis.

[7] Maia C., Dantas-Torres F., Campino L. (2018). Parasite biology: the reservoir hosts. *The Leishmaniases: Old Neglected Tropical Diseases*.

[8] PAHO–Pan American Health Organization (2024). Visceral leishmaniasis. https://www.paho.org/en/topics/leishmaniasis.

[9] Purse B. V., Masante D., Golding N. (2017). How will climate change pathways and mitigation options alter incidence of vector-borne diseases? A framework for leishmaniasis in South and Meso-America. *PLoS One*.

[10] Dantas-Torres F. (2007). The role of dogs as reservoirs of *Leishmania* parasites, with emphasis on *Leishmania (Leishmania) infantum* and *Leishmania (Viannia) braziliensis*. *Veterinary Parasitology*.

[11] Dantas-Torres F., Otranto D. (2016). Best practices for preventing vector-borne diseases in dogs and humans. *Trends in Parasitology*.

[12] Matsumoto P. S. S., Taniguchi H. H., Pereira V. B. R. (2022). Efficacies of insecticide dog collars against visceral leishmaniasis in low and high-income areas and the effects for non-collared neighbor dogs. *Acta Tropica*.

[13] Anversa L., Tiburcio M. G. S., Richini-Pereira V. B., Ramirez L. E. (2018). Human leishmaniasis in Brazil: a general review. *Revista da Associação Médica Brasileira*.

[14] Mann S., Frasca K., Scherrer S. (2021). A review of leishmaniasis: current knowledge and future directions. *Current Tropical Medicine Reports*.

[15] Soares P. H., da Silva E. S., Penaforte K. M. (2022). Responsible companion animal guardianship is associated with canine visceral leishmaniasis: an analytical cross-sectional survey in an urban area of southeastern Brazil. *BMC Veterinary Research*.

[16] Baneth G., Koutinas A. F., Solano-Gallego L., Bourdeau P., Ferrer L. (2008). Canine leishmaniosis–new concepts and insights on an expanding zoonosis: part one. *Trends in Parasitology*.

[17] Dantas-Torres F., Miró G., Baneth G. (2019). Canine leishmaniasis control in the context of one health. *Emerging Infectious Diseases*.

[18] Barbiéri C. L. (2006). Immunology of canine leishmaniasis. *Parasite Immunology*.

[19] Costa-da-Silva A. C., Nascimento D. O., Ferreira J. R. M. (2022). Immune responses in leishmaniasis: an overview. *Tropical Medicine and Infectious Disease*.

[20] Getnet M., Minaye Dejen A., Abebaw D., Fentahun G. G., Birhanu E. (2024). Diagnostic accuracy of serological rk-39 test for visceral leishmaniasis: systematic review and meta-analysis. *PLoS Neglected Tropical Diseases*.

[21] Reithinger R., Quinnell R. J., Alexander B., Davies C. R. (2002). Rapid detection of *Leishmania infantum* infection in dogs: comparative study using an immunochromatographic dipstick test, enzyme-linked immunosorbent assay, and PCR. *Journal of Clinical Microbiology*.

[22] Tkach M., Théry C. (2016). Communication by extracellular vesicles: where we are and where we need to go. *Cell*.

[23] Fernandez-Becerra C., Xander P., Alfandari D. (2023). Guidelines for the purification and characterization of extracellular vesicles of parasites. *Journal of Extracellular Biology*.

[24] Soares R. P., Xander P., Costa A. O. (2017). Highlights of the São Paulo ISEV workshop on extracellular vesicles in cross-kingdom communication. *Journal of Extracellular Vesicles*.

[25] Cortes-Serra N., Gualdron-Lopez M., Pinazo M. J., Torrecilhas A. C., Fernandez-Becerra C. (2022). Extracellular vesicles in *Trypanosoma cruzi* infection: immunomodulatory effects and future perspectives as potential control tools against Chagas disease. *Journal of Immunology Research*.

[26] Théry C., Ostrowski M., Segura E. (2009). Membrane vesicles as conveyors of immune responses. *Nature Reviews. Immunology*.

[27] Silverman J. M., Clos J., Horakova E. (2010). *Leishmania* exosomes modulate innate and adaptive immune responses through effects on monocytes and dendritic cells. *Journal of Immunology*.

[28] Silva V. O., Maia M. M., Torrecilhas A. C. (2018). Extracellular vesicles isolated fromToxoplasma gondiiinduce host immune response. *Parasite Immunology*.

[29] Carneiro F. M., Da Cruz A. B., Maia M. M. (2024). Extracellular vesicles from *Leishmania (Leishmania) infantum* contribute in stimulating immune response and immunosuppression in hosts with visceral leishmaniasis. *Microorganisms*.

[30] Dong G., Filho A. L., Olivier M. (2019). Modulation of host-pathogen communication by extracellular vesicles (EVs) of the protozoan parasite *Leishmania*. *Frontiers in Cellular and Infection Microbiology*.

[31] Atayde V. D., Aslan H., Townsend S., Hassani K., Kamhawi S., Olivier M. (2015). Exosome secretion by the parasitic protozoan *Leishmania* within the sand fly midgut. *Cell Reports*.

[32] Lira A., Fajardo E., Chang K., Clément P., Olivier M. (2022). *Leishmania* exosomes/extracellular vesicles containing GP63 are essential for enhance cutaneous leishmaniasis development upon co-inoculation of *Leishmania amazonensis* and its exosomes. *Frontiers in Cellular and Infection Microbiology*.

[33] Dong G., Wagner V., Minguez-Menendez A., Fernandez-Prada C., Olivier M. (2021). Extracellular vesicles and leishmaniasis: current knowledge and promising avenues for future development. *Molecular Immunology*.

[34] da Cruz A. B., Carneiro F. M., Maia M. M. (2023). Dogs with canine visceral leishmaniasis have a boost of extracellular vesicles and miR‐21‐5p up‐expression. *Parasite Immunology*.

[35] Laurenti M. D., de Santana Leandro M. V., Jr T. Y. (2014). Comparative evaluation of the DPP^®^ CVL rapid test for canine serodiagnosis in area of visceral leishmaniasis. *Veterinary Parasitology*.

[36] MSB-Ministerio da Saude do Brasil (2014). Manual de vigilância e controle da leishmaniose visceral (visceral leishmaniasis: surveillance and control. Technical manual). https://bvsms.saude.gov.br/bvs/publicacoes/manual_vigilancia_controle_leishmaniose_visceral_1edicao.pdf.

[37] Colombo F. A. (2012). *Detection of Leishmania (Leishmania) Infantum Chagasi RNA in Fleas and Ticks Collected From Naturally Infected Dogs and Standardization of a Real-Time PCR for Diagnosis and Differentiation of Leishmania Species, [Ph.D. thesis]*.

[38] Qvarnstrom Y., Schijman A. G., Veron V., Aznar C., Steurer F., da Silva A. J. (2012). Sensitive and specific detection of *Trypanosoma cruzi* DNA in clinical specimens using a multi-target real-time PCR approach. *PLoS Neglected Tropical Diseases*.

[39] Meira C. S., Vidal J. E., Costa-Silva T. A., Frazatti-Gallina N., Pereira-Chioccola V. L. (2011). Immunodiagnosis in cerebrospinal fluid of cerebral toxoplasmosis and HIV-infected patients using *Toxoplasma gondii* excreted/secreted antigens. *Diagnostic Microbiology and Infectious Disease*.

[40] Calculating & Analyzing ELISA data. https://www.assaygenie.com/calculating-analyzing-elisa-data/#Absorbance.

[41] How to Analyze ELISA Data. https://www.rndsystems.com/resources/how-to-analyze-elisa-data.

[42] Meira C. S., Vidal J. E., Costa-Silva T. A. (2013). IgG4 specific to *Toxoplasma gondii* excretory/secretory antigens in serum and/or cerebrospinal fluid support the cerebral toxoplasmosis diagnosis in HIV-infected patients. *Journal of Immunological Methods*.

[43] Cronemberger-Andrade A., Xander P., Soares R. P. (2020). *Trypanosoma cruzi*-infected human macrophages shed proinflammatory extracellular vesicles that enhance host-cell invasion via toll-like receptor 2. *Frontiers in Cellular and Infection Microbiology*.

[44] da Cruz A. B., Maia M. M., Pereira I. D. (2020). Human extracellular vesicles and correlation with two clinical forms of toxoplasmosis. *PLoS One*.

[45] Quiarim T. M., Maia M. M., da Cruz A. B., Taniwaki N. N., Namiyama G. M., Pereira-Chioccola V. L. (2021). Characterization of extracellular vesicles isolated from types I, II and III strains of *Toxoplasma gondii*. *Acta Tropica*.

[46] Hassani K., Antoniak E., Jardim A., Olivier M. (2011). Temperature-induced protein secretion by *Leishmania mexicana* modulates macrophage signalling and function. *PLoS One*.

[47] Tomiotto-Pellissier F., Bortoleti B. T. S., Assolini J. P. (2018). Macrophage polarization in leishmaniasis: broadening horizons. *Frontiers in Immunology*.

[48] Reis N. F. C., Dupin T. V., Costa C. R. (2020). *Leishmania amazo*nensis promastigotes or extracellular vesicles modulate B-1 cell activation and differentiation. *Frontiers in Cellular and Infection Microbiology*.

[49] Torrecilhas A. C., Soares R. P., Schenkman S., Fernández-Prada C., Olivier M. (2020). Extracellular vesicles in trypanosomatids: host cell communication. *Frontiers in Cellular and Infection Microbiology*.

[50] Marti M., Johnson P. J. (2016). Emerging roles for extracellular vesicles in parasitic infections. *Current Opinion in Microbiology*.

[51] Cota G. F., de Sousa M. R., de Freitas Nogueira B. M. (2013). Comparison of parasitological, serological, and molecular tests for visceral leishmaniasis in HIV-infected patients: a cross-sectional delayed-type study. *The American Journal of Tropical Medicine and Hygiene*.

[52] Georgiadou S. P., Makaritsis K. P., Dalekos G. N. (2015). Leishmaniasis revisited: current aspects on epidemiology, diagnosis and treatment. *Journal of Translational Internal Medicine*.

[53] Figueiredo M. M., Dos Santos A. R., Godoi L. C. (2021). Improved performance of ELISA and immunochromatographic tests using a new chimeric A2‐based protein for human visceral leishmaniasis diagnosis. *Journal of Immunology Research*.

[54] Cunningham J., Hasker E., Das P. (2012). A global comparative evaluation of commercial immunochromatographic rapid diagnostic tests for visceral leishmaniasis. *Clinical Infectious Diseases*.

[55] Sanchez M. C. A., Celeste B. J., Lindoso J. A. L. (2020). Performance of rK39-based immunochromatographic rapid diagnostic test for serodiagnosis of visceral leishmaniasis using whole blood, serum and oral fluid. *PLoS One*.

[56] Pereira I. E., Silva K. P., Menegati L. M. (2020). Performance of recombinant proteins in diagnosis and differentiation of canine visceral leishmaniasis infected and vaccinated dogs. *European Journal of Microbiology and Immunology*.

[57] Freire M. L., Machado de Assis T., Oliveira E. (2019). Performance of serological tests available in Brazil for the diagnosis of human visceral leishmaniasis. *PLoS Neglected Tropical Diseases*.

[58] Pedras M. J., de Gouvêa Viana L., de Oliveira E. J., Rabello A. (2008). Comparative evaluation of direct agglutination test, rK39 and soluble antigen ELISA and IFAT for the diagnosis of visceral leishmaniasis. *Transactions of the Royal Society of Tropical Medicine and Hygiene*.

[59] Pindedo-Cancino V., Kesper N., Barbiéri C. L., Lindoso J. A. L., Umezawa E. S. (2013). The efficacy of *L.* (*L.*) *chagasi* excreted-secreted antigens (ESAs) for visceral leishmaniasis diagnosis is due to low levels of cross-reactivity. *The American Journal of Tropical Medicine and Hygiene*.

[60] Saliba J. W., Lopes K. F., Silva-Pereira R. A., Teixeira L. A. S., Oliveira E. (2019). *Leishmania infantum* exo-antigens: application toward serological diagnosis of visceral leishmaniasis. *Parasitology Research*.

[61] Daltro R. T., Leony L. M., Freitas N. E. M. (2019). Cross-reactivity using chimeric *Trypanosoma cruzi* antigens: diagnostic performance in settings where Chagas disease and American cutaneous or visceral leishmaniasis are coendemic. *Journal of Clinical Microbiology*.

[62] Leony L. M., Freitas N. E. M., del-Rei R. P. (2019). Performance of recombinant chimeric proteins in the serological diagnosis of *Trypanosoma cruzi* infection in dogs. *PLoS Neglected Tropical Diseases*.

[63] Iturra J. A., Leony L. M., Medeiros F. A. (2023). A multicenter comparative study of the performance of four rapid immunochromatographic tests for the detection of anti-Trypanosoma cruzi antibodies in Brazil. *Frontiers in Medicine*.

[64] Fujimori M., de Almeida A. . B. P. F., Barrouin-Melo S. M. (2021). Validation of ELISA with recombinant antigens in serological diagnosis of canine *Leishmania infantum* infection. *Memórias Do Instituto Oswaldo Cruz*.

[65] Zanette M. F., Lima V. M., Laurenti M. D. (2014). Serological cross-reactivity of *Trypanosoma cruz*i, *Ehrlichia can*is, *Toxoplasma gondii, Neospora caninum* and *Babesia* canis to *Leishmania infantum chagasi* tests in dogs. *Revista da Sociedade Brasileira de Medicina Tropical*.

[66] Abass E., Kang C., Martinkovic F. (2015). Heterogeneity of *Leishmania donovani* parasites complicates diagnosis of visceral leishmaniasis: comparison of different serological tests in three endemic regions. *PLoS One*.

[67] Bhattacharyya T., Ayandeh A., Falconar A. K. (2014). IgG1 as a potential biomarker of post-chemotherapeutic relapse in visceral leishmaniasis, and adaptation to a rapid diagnostic test. *PLoS Neglected Tropical Diseases*.

[68] Marlais T., Bhattacharyya T., Singh O. P. (2018). Visceral leishmaniasis IgG1 rapid monitoring of cure vs. relapse, and potential for diagnosis of post kala-azar dermal leishmaniasis. *Frontiers in Cellular and Infection Microbiology*.

[69] de Lima C. M. F., Magalhães A. S., Costa R. (2021). High anti-Leishmania IgG antibody levels are associated with severity of mucosal leishmaniasis. *Frontiers in Cellular and Infection Microbiology*.

[70] Han X. Y., Li H. B., Wei J. H., Xu X. Y., Li Y., Che Y. Q. (2024). Serological characteristics and clinical implications of IgG subclasses in visceral leishmaniasis. *Tropical Medicine & International Health*.

[71] Quinnell R. J., Courtenay O., Garcez L. M. (2003). IgG subclass responses in a longitudinal study of canine visceral leishmaniasis. *Veterinary Immunology and Immunopathology*.

[72] Ribeiro P. A. F., Dias D. S., Lage D. P. (2018). A conserved *Leishmania* hypothetical protein evaluated for the serodiagnosis of canine and human visceral and tegumentary leishmaniasis, as well as a serological marker for the posttreatment patient follow-up. *Diagnostic Microbiology and Infectious Disease*.

[73] Chaabouni A., Elandoulsi R. B., Mhadhbi M., Gharbi M., Sassi A. (2018). Comparative analysis of the Leishmania infantum-specific antibody repertoires and the autoantibody repertoires between asymptomatic and symptomatic dogs. *Veterinary Parasitology*.

[74] Olías-Molero A. I., Moreno I., Corral M. J. (2020). Infection of dogs by *Leishmania infantum* elicits a general response of IgG subclasses. *Scientific Reports*.

